# Costs of Chronic Diseases at the State Level: The Chronic Disease Cost Calculator

**DOI:** 10.5888/pcd12.150131

**Published:** 2015-09-03

**Authors:** Justin G. Trogdon, Louise B. Murphy, Olga A. Khavjou, Rui Li, Christopher M. Maylahn, Florence K. Tangka, Tursynbek A. Nurmagambetov, Donatus U. Ekwueme, Isaac Nwaise, Daniel P. Chapman, Diane Orenstein

**Affiliations:** Author Affiliations: Louise B. Murphy, Daniel P. Chapman, Division of Population Health, Centers for Disease Control and Prevention, Atlanta, Georgia; Olga A. Khavjou, RTI International, Research Triangle Park, North Carolina; Rui Li, Division of Diabetes Translation, Centers for Disease Control and Prevention, Atlanta, Georgia; Christopher M. Maylahn, New York State Department of Health, Albany, New York; Florence K. Tangka, Donatus U. Ekwueme, Division of Cancer Prevention and Control, Centers for Disease Control and Prevention, Atlanta, Georgia; Tursynbek A. Nurmagambetov, Division of Environmental Hazards and Health Effects, Centers for Disease Control and Prevention, Atlanta, Georgia; Isaac Nwaise, Division for Heart Disease and Stroke Prevention, Centers for Disease Control and Prevention, Atlanta, Georgia; Diane Orenstein, Division of Community Health, Centers for Disease Control and Prevention, Atlanta, Georgia.

## Abstract

**Introduction:**

Many studies have estimated national chronic disease costs, but state-level estimates are limited. The Centers for Disease Control and Prevention developed the Chronic Disease Cost Calculator (CDCC), which estimates state-level costs for arthritis, asthma, cancer, congestive heart failure, coronary heart disease, hypertension, stroke, other heart diseases, depression, and diabetes.

**Methods:**

Using publicly available and restricted secondary data from multiple national data sets from 2004 through 2008, disease-attributable annual per-person medical and absenteeism costs were estimated. Total state medical and absenteeism costs were derived by multiplying per person costs from regressions by the number of people in the state treated for each disease. Medical costs were estimated for all payers and separately for Medicaid, Medicare, and private insurers. Projected medical costs for all payers (2010 through 2020) were calculated using medical costs and projected state population counts.

**Results:**

Median state-specific medical costs ranged from $410 million (asthma) to $1.8 billion (diabetes); median absenteeism costs ranged from $5 million (congestive heart failure) to $217 million (arthritis).

**Conclusion:**

CDCC provides methodologically rigorous chronic disease cost estimates. These estimates highlight possible areas of cost savings achievable through targeted prevention efforts or research into new interventions and treatments.

## Introduction

Chronic diseases lead to increased medical costs and productivity losses (eg, time lost from work) (1–4). Costs attributable to chronic disease are expected to continue to grow (5). Many studies have estimated national chronic disease costs, but state-level estimates are limited. The Centers for Disease Control and Prevention (CDC) developed the Chronic Disease Cost Calculator (CDCC) in response to state chronic disease programs’ requests for technical assistance in estimating state-level chronic disease costs. The CDCC was created in partnership with the National Association of Chronic Disease Directors, the Agency for Healthcare Research and Quality (AHRQ), and the National Pharmaceutical Council (6). In this article, we provide an overview of CDCC. Additional details, including the software and technical appendix, are available at www.cdc.gov/chronicdisease/calculator/ (6).

The first version of the CDCC, released in 2009, estimated state-level Medicaid costs for 6 chronic diseases (6). The second version, released in 2013 and described in this article, provides (new features in *italics*) state-level medical costs, *absenteeism costs*, and *10-year medical cost projections* for *arthritis, asthma,* cancer, congestive heart failure (CHF), coronary heart disease (CHD), hypertension, stroke, other heart diseases, *depression,* and diabetes (Table 1). Medical estimates are generated for the entire state population (*all payers* including the uninsured) and separately for those covered by specific payers (Medicaid, *Medicare*, and *private insurance)*. For each state, payer, chronic disease, and subpopulation selected, the CDCC generates a standardized report presenting the number of people treated for the disease, annual payer and absenteeism costs attributable to the disease (per-person and total), and 10-year medical cost projections for all payers combined. These estimates supplement other disease burden measures of chronic disease impact (eg, prevalence) and may identify opportunities for targeting prevention programs to reduce risk factors and chronic diseases. CDCC users can also generate customized state-level cost estimates for selected chronic diseases by modifying key inputs. For example, the financial impact of prevention programs could be simulated by inputting treated population prevalence from other sources. The purpose of this article is to demonstrate the CDCC by providing an example across all states and all payers.

**Table 1:** Chronic Disease Definitions, Clinical Classification System Code^a^ and 3-Digit ICD-9-CM

Disease	Code
**Arthritis**	ICD-9-CM: 274, 354, 390, 391, 443, 446, 710–716, 719–721, 725–729
**Asthma**	CCS: 128; ICD-9-CM: 493
**Cancer**	CCS: 11–43, 45
**Cardiovascular disease**	
Diseases of the heart	
Congestive heart failure	ICD-9-CM: 428
Coronary heart disease	ICD-9-CM: 410–414
Other heart diseases^b^	ICD-9-CM: 390–392, 393–398, 415–416, 420–427, 429
Hypertension	ICD-9-CM: 401–405
Stroke	ICD-9-CM: 430–434, 436–438
**Depression**	ICD-9-CM: 296, 311
**Diabetes**	ICD-9-CM: 250

Abbreviations: CCS, Clinical Classification System; ICD-9-CM, International Classification of Diseases, 9th revision, clinical modification.

a CCS codes collapse ICD-9-CM codes into a smaller number of clinically meaningful categories (14).

b Includes arrhythmias, bacterial endocarditis, cardiomyopathy, congenital cardiovascular defects, rheumatic fever/rheumatic heart disease, and valvular heart disease.

## Methods

The CDCC calculates state-level chronic disease medical and absenteeism costs using 4 components: 1) state-specific payer populations, 2) treated population prevalence, 3) per-person medical and absenteeism costs, and 4) medical cost projections (7). The costs are representative of the noninstitutionalized and nursing home population.

### Component 1: state-specific payer populations

Default state-specific payer populations (ie, number with health care coverage through payer) were estimated for all payers combined and by specific payer category. Payer populations were not mutually exclusive (eg, persons dually eligible for Medicare and Medicaid were in each payer population). Payer populations were calculated by using the following data: 2008 US Census Bureau for all payers combined, fiscal year 2008 Medicaid Statistical Information Statistics for Medicaid, Kaiser Family Foundation 2008 Medicare Health and Prescription Drug Plan Tracker for Medicare, and 2008 Current Population Survey (CPS) for the privately insured (8–12).

For each payer group, we estimated the proportion in each of the 6 unique age (18–44, 45–64, or ≥65 years) and sex groups. We included children (ie, aged 0 to 17) for asthma and depression only because these conditions are common among them. Age and sex distribution for all payers and private payers were calculated using 2008 US Census Bureau and 2008 CPS data, respectively. We estimated Medicaid and Medicare enrolment by age and sex using 2007 through 2009 CPS data. Unlike the CMS population counts, CPS represents the noninstitutionalized population only. We derived each age- and sex-specific state-level payer population by multiplying state payer populations by the age- and sex-specific proportions.

### Component 2: treated population prevalence

Treated population prevalence (ie, percentage treated annually) was estimated in 2 steps. In step 2A, we estimated treated prevalence for the noninstitutionalized population using data from the 2004–2008 Medical Expenditure Panel Survey (MEPS) (13). MEPS is a nationally representative survey of the civilian noninstitutionalized population administered by AHRQ. Professional coders transcribe self-reported MEPS condition data using the *International Classification of Diseases, Ninth Revision, Clinical Modification* (ICD-9-CM). We defined each disease using codes from the AHRQ Clinical Classification System or CDC definitions (both based on ICD-9-CM codes) (Table 1) (14). Treated prevalence was receipt of any medical care, including prescription drugs, in the previous year.

For each payer population in the noninstitutionalized population, we used separate logistic regressions to predict the probability of having each disease (4 payers × 10 chronic conditions = 40 models). For each individual *i* in payer population *p*, we estimated the probability of disease *d*:

*Pr*(*Y*_ipd_ = 1) = Λ(Age_ip_, Sex_ip_, Region_ip_, Year_ip_)

These models controlled for survey year and survey participants’ age, sex, and region of residence (Northeast, South, Midwest, or West). All regression models accounted for MEPS’ complex survey design. We identified significant age-by-sex-by-region interactions (at α = .05) using stepwise regressions. We predicted the nationally representative treated population prevalence for 2004 through 2008 from the final, survey-weighted logistic regressions for each age, sex, and region combination (2A).

Because MEPS does not survey the noninstitutionalized population, we adjusted estimates by the most recent (2004) National Nursing Home Survey (NNHS) (nationally representative sample of US nursing homes) so that costs include treatment received by nursing home residents (step 2B) (15). CDCC does not include other institutionalized and noncivilian populations. We defined diseases using ICD-9-CM codes based on any condition diagnosis (primary or secondary) at either time of admission or survey (Table 1). For each payer (payment source in NNHS), age, and sex group, we used a survey-weighted total of the number of people with each disease and scaled the MEPS treated population prevalence by the following ratio: number with disease (MEPS + NNHS) / number with disease (MEPS).

We calculated the number who were treated for each disease in 2008 by multiplying the estimated treatment population prevalence for each payer/age/sex/region group by the estimated number in the corresponding payer/age/sex/state specific category from Component 1 (Table 2).

**Table 2:** Example of Components of All Payer Medical Cost Estimates for Coronary Heart Disease in California

Item	Component No.	Estimate
Population	1	36,756,666
Percentage treated	2	3.1
Treated population	2	1,156,300
Cost per person, $	3	6,800
Total cost, $, millions	** —**	7,857

Abbreviation: —, not applicable.

### Component 3: medical and absenteeism costs

#### Medical Costs

“All payers” analysis contained all medical costs in the 2004–2008 MEPS (payments, not providers’ charges, by public and private insurers and out-of-pocket payments by treatment recipients or other noninsurance payers). Each payer’s analysis used only costs paid by that payer.

For each payer, we estimated default chronic disease medical costs using a 5-part process. In step 3A(i), we estimated per-person medical costs using a logistic regression model that predicted the probability that a person incurred any medical costs and a generalized linear model with a gamma distribution and log link that estimated annual medical costs for those incurring such costs.

Hypertension and diabetes are risk factors for other chronic conditions, and costs for these conditions were estimated in separate models that did not control for these sequelae conditions; that is, the hypertension and diabetes models did not control for CHF, CHD, stroke, other heart diseases, and renal failure. Additionally, the diabetes model did not adjust for hypertension and dyslipidemia. Therefore, the CDCC’s default estimates for hypertension and diabetes include the costs of sequelae. We caution users that summing costs across selected diseases that include hypertension and diabetes will overestimate costs for all the selected diseases. The CDCC provides estimates for diseases of the heart and total cardiovascular disease but avoids double-counting of costs across diseases using methods described below (step 3A[ii]).

In step 3A(ii), we calculated each disease’s attributable costs by comparing predicted costs for people with each unique combination of diseases with predicted costs for people without that combination of diseases, while holding all other characteristics constant. For example, we calculated costs of treated cancer alone and cancer with hypertension as 2 different disease combinations. Then we divided the total costs attributable to the disease combinations back to the constituent diseases (ie, share of all cancer with hypertension costs that are attributable to cancer). In this combination of diseases, the greater share of the costs is apportioned to the disease with the larger regression coefficient (16). We estimated per-person medical costs for each disease and age/sex/region category using the national model’s coefficients (step 3A[i]).

In step 3A(iii), we used 2004 NNHS (15) and National Health Expenditure Accounts (17) data to adjust the per-person medical costs to account for treatment received by nursing home residents while in a nursing home (7). Because public use data on nursing home residents’ disease treatment costs outside a nursing home were not available, we assumed these costs were the same as per-person medical costs for the noninstitutionalized population.

In step 3A(iv), we used restricted-access MEPS data to generate state-specific cost adjustments for the 30 largest states and 9 Census divisions for the smallest 20 states and the District of Columbia. Among respondents with any medical costs, we regressed medical costs (on a log scale) on state/census division and adjustment variables (technical appendix) using a 1-part model. We estimated state-specific estimates, we multiplied the per-person costs from step 3A(iii) by the coefficients from step 3A(iv). For example, if per-person costs from the national model 3A(iii) were $1,000 and the state coefficient from 3A(iv) was 1.10, state-specific per-person costs were $1,100 ($1,000 × 1.10).

In step 3A(v), we adjusted MEPS cost data to 2010 dollars using the gross domestic product price deflator (18) and multiplied per-person medical cost estimates in each age/sex category by the estimated number of people covered by each payer in each state from component one.

#### Absenteeism Costs

We separately estimated workdays lost for working adults and implied lost workdays for parents of children who lost schooldays. We predicted annual workdays missed by working adults using a negative binomial model. The dependent variable was annual workdays missed due to illness or injury. Independent variables were identical to medical cost regressions, including use of alternate models for hypertension and diabetes. Among children, we estimated annual number of school days missed due to illness or injury using negative binomial models. We assumed that one working parent also misses work on the days that his or her child is absent from school. We calculated workdays missed per person attributable to each disease using the same method as that used for medical costs (16). We estimated the value of missed workdays using age/sex-specific average daily earnings (not including fringe benefits) from the 2009 CPS.

### Component 4: medical cost projections

We calculated projected chronic disease medical costs for all payers combined through 2020 using default cost estimates and projected 2010 through 2020 US Census Bureau state population counts (9). For each state, we multiplied the treated population prevalence (age- and sex-specific; component 2) by projected number of state residents in the corresponding category for each year (2010 through 2020). Then we summed these age- and sex-specific estimates to project the total number treated for each disease by state and year. We multiplied the projected per-person cost of each disease for people in each age/sex/state category (component 3) by the number in the corresponding category that were estimated to be treated for that disease. We summed the projections for each category to estimate total annual costs of care for each disease. Finally, we adjusted the cost projections for the Congressional Budget Office’s estimate of real growth in medical spending not attributable to population growth and aging (19). Projections do not include cost increases due to inflation. Projections assume no changes in insurance coverage or technology (20) and that all of changes in the medical costs are driven by medical cost growth (19) and changes in population size and demographics. The technical appendix contains additional details on the analysis (12).

## Results

Among the 10 specific conditions reported in the CDCC, median state-specific medical costs ranged from $410 million for asthma to $1.8 billion for diabetes; median absenteeism costs ranged from $5 million for CHF to $217 million for arthritis (Table 3). Across states and chronic conditions, Medicaid and Medicare medical costs represented 29% (depression) to 57% (CHF) of overall state medical costs.

**Table 3:** State Costs Attributable to Selected Chronic Diseases, in Millions, 2010 Dollars

Chronic Condition	Medical Costs	Absenteeism Costs	Medicaid and Medicare’s Portion of State Costs, % (Range)
	Median (Range)		
**Arthritis**	1,623 (206–11,490)	217 (28–1,791)	34.5 (29.1–42.5)
**Asthma**	410 (55–3,182)	48 (6–383)	50.3 (33.6–72.7)
**Cancer**	1,973 (227–13,614)	116 (15–916)	37.1 (30.4–43.0)
**Cardiovascular disease, total**	4,302 (411–26,062)	201 (23–1,396)	42.2 (37.6–51.7)
Diseases of the heart	2,376 (211–14,313)	76 (8–487)	45.8 (41.3–54.0)
Congestive heart failure	256 (18–1,702)	5 (0–42)	56.7 (47.4–72.4)
Coronary heart disease	1,340 (18–7,988)	58 (6–383)	38.9 (33.9–45.5)
Other heart diseases	765 (68–4,623)	13 (1–79)	55.5 (47.3–68.2)
Hypertension	1,624 (167–10,032)	78 (9–546)	35.4 (30.8–45.8)
Stroke	873 (86–5,959)	64 (7–451)	50.9 (41.6–67.4)
**Depression**	892 (120–6,728)	97 (13–861)	29.0 (23.1–37.9)
**Diabetes**	1,780 (212–12,095)	72 (9–544)	40.1 (32.9–50.1)

## Discussion

Public health officials often lack the resources to estimate the economic burden of chronic diseases. The CDCC estimates medical and absenteeism costs of chronic diseases without additional data requirements. Decision makers can generate customized estimates for local populations of interest using accurate local data for these populations.

The CDCC’s default estimates may differ from other cost estimates because of variation in data sources, settings, populations studied, disease prevalence definitions, cost measurement, and study methods. For example, the Behavioral Risk Factor Surveillance System (BRFSS) prevalence definition is based on a lifetime disease history (21) whereas the CDCC population prevalence required treatment within the year. Thus, the CDCC likely contains higher costs but fewer persons with disease. Combining disease prevalence estimates from other sources with MEPS per-person treatment costs could overestimate total costs. Therefore, we recommend using CDCC’s treated disease prevalence estimates.

CDCC estimates may differ from other chronic disease cost estimates reported elsewhere. There is substantial heterogeneity in methods used to derive medical cost estimates, and the most appropriate approach to estimating disease costs depends on the users’ needs, available data sources, and cost definitions. Some costs are based on summing of payments only for claims with diagnosis codes for the disease of interest (2,3) or summing all medical costs for persons with the disease (1). CDCC aims to minimize duplicated costs by estimating costs for persons with the disease that are attributed to the disease. Different methods have been used to attribute costs to diseases (22,23) to avoid double counting of costs across diseases (16) and to adjust for potential confounders. CDCC estimates may also differ from claims-based analysis, especially because CDCC estimates were derived from multiple data sources based on data from different geographic units (region, nation), and results from the statistical analysis are themselves estimates. MEPS underestimates total medical expenditures because 1) it does not capture all medical care use and expenditures, including nondurable goods (eg, over-the-counter medications) and health services administered in nonmedical settings (24); and 2) comparison of MEPS with other medical use data demonstrates that MEPS respondents underreport medical events, which leads to underestimates of treated prevalence and use (25).

CDCC results are not comparable across states for at least 2 reasons. First, they were derived from data from different levels of geographic detail (eg, methods for the 30 largest states differed from those for smaller states), and second, MEPS survey weights are not designed to be representative of state populations. CDCC does not estimate productivity losses through caregiving to adults with chronic disease, impaired productivity while at work, premature mortality, and reductions in the quality of life.

CDCC’s medical cost projections reflect historical medical cost growth and demographic changes but do not account for changes from Patient Protection and Affordable Care Act implementation. However, projections provide a useful baseline for gauging impact of current and future policies and may inform budgetary allocations for chronic disease prevention and early detection investments.

The CDCC provides state-level estimates of the economic burden of selected chronic diseases, because many policy decisions are made at the state level. Estimates highlight possible areas of cost savings achievable through targeted prevention efforts or research into new interventions and treatments. This information is vital for program decisions and resource allocations for chronic disease prevention and disease management programs.
